# 基于二代测序的微小残留病定量分层对诱导治疗后初治多发性骨髓瘤早期疾病进展的预测价值

**DOI:** 10.3760/cma.j.cn121090-20260403-00173

**Published:** 2026-07

**Authors:** 雨荷 彭, 迅 周, 霜 颜, 利 姚, 德沛 吴, 琤琤 傅

**Affiliations:** 苏州大学附属第一医院血液科，血液学协同创新中心，国家血液系统疾病临床医学研究中心，江苏省血液研究所，苏州 215006 National Clinical Research Center for Hematologic Diseases, Jiangsu Institute of Hematology, Collaborative Innovation Center of Hematology, the First Affiliated Hospital of Soochow University, Suzhou 215006, China

**Keywords:** 多发性骨髓瘤, 肿瘤，残余, 二代测序, 诱导治疗, 造血干细胞移植, Multiple myeloma, Neoplasm, residual, Next-generation sequencing, Induction therapy, Hematopoietic stem cell transplantation

## Abstract

**目的:**

评估基于二代测序（NGS）的微小残留病（MRD）定量分层对诱导治疗后初治多发性骨髓瘤（NDMM）患者早期疾病进展的预测价值。

**方法:**

本回顾性队列研究分析了2019年至2024年苏州大学附属第一医院血液科收治的408例接受VRD方案（硼替佐米+来那度胺+地塞米松）诱导治疗联合自体造血干细胞移植（auto-HSCT）的NDMM患者的临床资料。于4个疗程诱导结束后、auto-HSCT前采集患者的骨髓标本，采用灵敏度10^−6^的NGS技术检测MRD。按MRD负荷将患者分为超深度阴性组（<10^−6^）、低负荷阳性组（≥10^−6^且≤10^−3^）及高负荷残留组（>10^−3^），比较各组患者的临床特征及预后。

**结果:**

4个疗程VRD方案诱导治疗后患者总反应率为96.8％（395/408），疗效达完全缓解及以上197例（48.3％）。超深度阴性组（90例）、低负荷阳性组（142例）、高负荷残留组（176例）del（17p）阳性率分别为4.4％、15.5％、10.8％（*P*＝0.030）；t（4;14）阳性率分别为13.3％、19.0％、28.4％（*P*＝0.021），以高负荷残留组最高。超深度阴性组的疾病进展风险较高负荷残留组降低86％（*HR*＝0.14，95％ *CI*：0.08～0.26，*P*<0.001）。超深度阴性组、低负荷阳性组及高负荷残留组24个月累积进展发生率（CIF）分别为1.2％（95％ *CI*：0.1％～5.9％）、8.3％（95％ *CI*：4.4％～13.9％）、23.2％（95％ *CI*：17.2％～29.7％）。以高负荷残留组为参照，超深度阴性组（*sHR*＝0.15，95％ *CI*：0.08～0.31）及低负荷阳性组（*sHR*＝0.33，95％ *CI*：0.22～0.50）疾病进展风险均显著降低（*P*值均<0.001）；超深度阴性组疾病进展风险较低负荷阳性组亦显著降低（*sHR*＝0.48，95％ *CI*：0.24～0.94，*P*＝0.032）。多因素分析显示，MRD水平为独立预后因素，高负荷残留组的疾病进展风险较超深度阴性组显著升高（*sHR*＝6.65，95％ *CI*：3.58～12.33，*P*<0.001）。

**结论:**

基于NGS的MRD定量分层对诱导治疗后NDMM患者早期疾病进展有独立预测价值。

## Abstract (en)

**Objective:**

To evaluate the predictive value of next-generation sequencing（NGS）-based quantitative minimal residual disease（MRD）stratification for early disease progression in patients newly diagnosed with multiple myeloma（NDMM）after induction therapy.

**Methods:**

This retrospective cohort study analyzed the clinical data of 408 patients with NDMM admitted to the Department of Hematology, the First Affiliated Hospital of Soochow University between 2019 and 2024, who received the bortezomib + lenalidomide + dexamethasone（VRD）regimen induction therapy combined with autologous hematopoietic stem cell transplantation（auto-HSCT）. Bone marrow samples were collected after 4 cycles of induction therapy and before auto-HSCT, and MRD was detected by NGS technology with a sensitivity of 10–6. Patients were divided into the ultra-deep negative group（<10^−6^）, low-load positive group（≥10^−6^ and ≤10^−3^）, and high-load residual group（>10^−3^）according to MRD burden, and the clinical characteristics and prognosis of each group were compared.

**Results:**

After cycles of VRD induction therapy, the overall response rate was 96.8%（395/408）, and 197 patients（48.3％）achieved complete remission or better. The positive rates of del（17p）were 4.4％, 15.5％, and 10.8％, respectively（*P*＝0.030）and the positive rates of t（4;14）were 13.3％, 19.0％, and 28.4％（*P*＝0.021）in the ultra-deep negative group（90 patients）, low-load positive group（142 patients）, and high-load residual group（176 patients）, respectively, with the highest rate observed in the high-load residual group. The risk of disease progression in the ultra-deep negative group was reduced by 86％ compared with the high-load residual group（*HR*＝0.14, 95％*CI*: 0.08–0.26, *P*<0.001）. The 24-month cumulative incidence of progression（CIF）in the ultra-deep negative group, low-load positive group, and high-load residual group was 1.2％（95％ *CI*: 0.1％–5.9％）, 8.3％（95％ *CI*: 4.4％–13.9％）, and 23.2％（95％ *CI*: 17.2％–29.7％）, respectively. Using the high-load residual group as reference, the risk of disease progression was significantly reduced in both the ultra-deep negative group（*sHR* = 0.15, 95％ *CI*: 0.08–0.31) and the low-load positive group（*sHR*＝0.33, 95％ *CI*: 0.22–0.50）（both *P*<0.001）. The risk of disease progression in the ultra-deep negative group was also significantly lower than that in the low-load positive group（sHR＝0.48, 95％*CI*: 0.24–0.94, *P*＝0.032）. Multivariate analysis revealed that the MRD level was an independent prognostic factor, and the risk of disease progression in the high-load residual group was significantly higher than that in the ultra-deep negative group（*sHR*＝6.65, 95％*CI*: 3.58–12.33, *P*<0.001）.

**Conclusion:**

NGS-based quantitative MRD stratification has independent predictive value for early disease progression in patients with NDMM after induction therapy.

多发性骨髓瘤（MM）是一种克隆浆细胞异常增殖的恶性疾病，发病率居血液系统恶性肿瘤第二位[1]。受老龄化社会发展的影响，我国MM的发病率正逐年升高[2]。尽管蛋白酶体抑制剂及免疫调节剂等新药的临床应用改善了MM患者的预后，但疾病仍会复发，实现持久、有效的临床缓解成为当前的治疗目标[3]–[5]。临床缓解率不仅是评价药物疗效的指标，更是影响患者长期生存的关键预后因素。微小残留病（MRD）检测代表了超越传统疗效评估的分子层面缓解情况评价，其对生存结局的改善作用可能独立于基线特征和治疗方案[4],[6]–[9]。多项荟萃分析及临床试验已证实，MRD阴性状态与MM患者无进展生存（PFS）期及总生存（OS）期的显著改善密切相关，MRD已逐渐成为替代传统疗效评估标准的重要预后生物标志物[9]–[11]。

二代测序（NGS）凭借其高灵敏度和标准化的优势，成为MRD评估的核心技术之一[12]–[14]，为实现更精细的MRD分层提供了技术基础。MRD状态不仅具有预后判断价值，更有望成为调整治疗强度的动态生物标志物[15]–[17]。对于接受VRD方案（硼替佐米+来那度胺+地塞米松）联合自体造血干细胞移植（auto-HSCT）一线标准治疗方案的患者，在诱导治疗结束这一早期节点是否可以通过精细的定量分层识别高危复发患者以调整后续治疗强度，目前尚缺乏大规模临床证据。本研究回顾性分析408例接受VRD方案诱导治疗序贯auto-HSCT的初治MM患者，采用NGS（灵敏度为10^−6^）对诱导治疗后MRD负荷进行三级定量分层，明确其对早期疾病进展的预测价值，为临床个体化风险分层与治疗决策优化提供循证依据。

## 病例与方法

1. 病例：本回顾性队列研究纳入2019年7月至2024年12月在苏州大学附属第一医院血液科接受治疗的789例初治多发性骨髓瘤（NDMM）患者。纳入标准：①符合《中国多发性骨髓瘤诊治指南（2024年修订）》[18]定义的NDMM诊断标准；②接受VRD方案诱导治疗序贯auto-HSCT；③诱导治疗后有完整NGS检测数据及随访资料。排除标准：诊断为浆细胞白血病或原发性淀粉样变性、合并其他活动性恶性肿瘤、基线或MRD监测数据缺失者。最终纳入408例患者。本研究经苏州大学附属第一医院伦理委员会批准（批件号：2025伦研批第1012号），所有患者均知情同意。

2. 治疗方案：所有患者接受4个周期VRD方案诱导治疗（每个周期28 d）：硼替佐米1.3 mg/m^2^皮下注射（第1、4、8、11天），来那度胺25 mg/d口服（第1～14天，其中4.2％的患者因肾功能损害根据肌酐清除率将剂量调整至10 mg/d），地塞米松20 mg静脉注射（第1～2、4～5、8～9、11～12天）；所有患者硼替佐米及地塞米松均按照标准方案给药。诱导治疗结束后予大剂量美法仑（200 mg/m^2^）预处理序贯auto-HSCT，此后酌情行2个周期VRD方案巩固治疗。维持治疗依据细胞遗传学风险分层：标准危险患者接受来那度胺单药维持；高危细胞遗传学［FISH检测t（4;14）、t（14;16）或del（17p）阳性］或R-ISS Ⅲ期患者接受来那度胺联合硼替佐米维持治疗至疾病进展[19]。

3. MRD检测方法：于VRD方案诱导治疗结束后（第4个疗程治疗结束后4周内）采集骨髓标本，采用基于NGS的方法进行MRD定量检测，具体流程如下：EDTA抗凝骨髓抽吸液经红细胞裂解及离心获取有核细胞，采用Maxwell®自动化DNA纯化系统（美国Promega公司）提取基因组DNA，经Qubit® 2.0荧光定量仪精确定量。为确保检测灵敏度达10^−6^，每个样本DNA起始投入量不低于3 000 ng。采用基于扩增子的方法构建NGS文库，利用LymphoTrack® IGH检测系统（美国Invivoscribe公司）靶向IGH-FR1/FR2/FR3框架，扩增程序为95 °C初始变性7 min，29个循环（95 °C 45 s、60 °C 45 s、72 °C 90 s），终延伸72 °C 10 min。扩增产物经AMPure® XP磁珠（美国贝克曼库尔特有限公司产品）纯化后定量，文库标准化至12～20 pmol/L后于Ion S5™测序系统（美国赛默飞世尔科技公司）完成测序。原始数据经LymphoTrack®分析软件v1.1.0（美国Invivoscribe公司）处理，通过追踪诊断基线克隆性重排序列实现MRD定量评估。检测流程及结果报告参照相关专家共识[20]–[21]。

4. 研究终点：PFS期定义为自患者诊断至疾病进展或因任何原因死亡的时间，OS期定义为自患者诊断至因任何原因死亡的时间。累积进展发生率（CIF）定义为在考虑竞争风险事件（非疾病进展相关死亡）条件下估计疾病进展的累积概率。疾病进展模式分为两类：单纯生化进展和临床进展。单纯生化进展定义为仅出现M蛋白或轻链水平升高，达到国际骨髓瘤工作组（IMWG）的疾病进展标准[12]。临床进展是出现新发骨骼破坏、贫血、高钙血症或肾功能不全等症状或器官损害，或出现新发髓外病变，包括软组织浆细胞瘤或浆细胞浸润至髓外器官/组织。

5. 随访和疗效评估：通过查阅电子病历系统及电话联系进行随访，随访截止日期为2025年9月。疗效评估依据IMWG标准[12]，分为严格意义的完全缓解、完全缓解（CR）、非常好的部分缓解（VGPR）、部分缓解（PR）、疾病稳定及疾病进展。总反应率（ORR）定义为疗效达PR及以上患者的比例。

6. 统计学处理：分类变量以例数（百分比）表示，组间差异采用卡方检验或Fisher精确检验。连续变量以*M*（*Q*_1_，*Q*_3_）或*M*（范围）表示，组间比较采用Kruskal-Wallis检验。生存分析采用Kaplan-Meier法，组间比较采用Log-rank检验。CIF组间差异比较采用Gray检验。鉴于队列中存在非疾病进展相关死亡，采用Fine-Gray竞争风险回归模型对疾病进展的独立预后因素进行多因素分析[22]。多重比较采用Benjamini-Hochberg法校正假阳性率。双侧*P*<0.05为差异有统计学意义。所有统计分析采用R软件（v4.4.1）的survival、survminer及cmprsk程序包完成。

## 结果

1. 临床特征：本研究共纳入408例NDMM患者，男232例（56.9％），中位年龄56.0（49.0，61.0）岁。免疫分型以IgG型最常见（47.5％，194/408），其次为轻链型（29.9％，122/408）和IgA型（17.2％，70/408）。疾病分期方面，DS分期Ⅲ期患者371例（90.9％），R-ISS分期Ⅲ期66例（16.2％）。细胞遗传学异常包括t（4;14）（21.8％，89/408）、t（14;16）（1.2％，5/408）及del（17p）（11.0％，45/408），另外219例（53.7％）患者携带1q21获得/扩增。随访期间138例（33.8％）发生疾病进展，66例（16.2％）死亡，其中18例为非疾病进展相关死亡。

根据诱导治疗后的MRD水平将患者分为三组：超深度阴性组（<10^−6^）、低负荷阳性组（≥10^−6^且≤10^−3^）、高负荷残留组（>10^−3^）。三组患者性别、年龄、M蛋白分型、分期及主要实验室指标的差异均无统计学意义（*P*值均>0.05）（表1）。细胞遗传学方面，三组患者del（17p）和t（4;14）发生率的差异均有统计学意义（*P*值分别为0.030和0.021）（表1）。

**表1:** 超深度阴性组、低负荷阳性组、高负荷残留组初治多发性骨髓瘤患者的基线特征比较

特征	超深度阴性组（90例）	低负荷阳性组（142例）	高负荷残留组（176例）	*χ*^2^/*H*值	*P*值
男性［例（％）］	51（56.7）	74（52.1）	107（60.8）	2.417	0.294
年龄［岁，*M*（*Q*_1_，*Q*_3_）］	57.0（50.0，63.0）	55.0（49.0，60.0）	56.0（51.0，62.0）	1.696	0.431
分型［例（％）］				–	0.238
IgA型	19（21.1）	19（13.4）	32（18.2）		
IgG型	37（41.1）	73（51.4）	84（47.7）		
IgD型	0（0）	4（2.8）	8（4.5）		
轻链型	31（34.4）	42（29.6）	49（27.8）		
其他	3（3.3）	4（2.8）	3（1.7）		
DS分期［例（％）］				1.416	0.493
Ⅰ、Ⅱ期	11（12.2）	12（8.5）	14（8.0）		
Ⅲ期	79（87.8）	130（91.5）	162（92.0）		
ISS分期［例（％）］				3.179	0.204
Ⅰ、Ⅱ期	66（73.3）	92（64.8）	110（62.5）		
Ⅲ期	24（26.7）	50（35.2）	66（37.5）		
R-ISS分期［例（％）］				0.266	0.879
Ⅰ、Ⅱ期	77（85.6）	118（83.1）	147（83.5）		
Ⅲ期	13（14.4）	24（16.9）	29（16.5）		
1q21［例（％）］				0.251	0.882
阴性	41（45.6）	64（45.1）	84（47.7）		
获得/扩增	49（54.4）	78（54.9）	92（52.3）		
del（17p）［例（％）］	4（4.4）	22（15.5）	19（10.8）	6.870	0.030
t（4;14）［例（％）］	12（13.3）	27（19.0）	50（28.4）	7.726	0.021
t（11;14）［例（％）］	8（8.9）	11（7.7）	26（14.8）	4.816	0.090
t（14;16）［例（％）］	2（2.2）	1（0.7）	2（1.2）	–	0.599
HGB［g/L，*M*（*Q*_1_，*Q*_3_）］	93.00（74.00，113.00）	98.50（84.00，116.50）	95.00（75.00，115.00）	2.123	0.346
PLT［×10^9^/L，*M*（*Q*_1_，*Q*_3_）］	148.00（131.00，212.00）	177.00（135.00，224.25）	162.00（130.00，205.00）	2.242	0.326
钙［mmol/L，*M*（*Q*_1_，*Q*_3_）］	2.24（2.14，2.32）	2.30（2.17，2.45）	2.28（2.16，2.46）	4.396	0.111
β_2_-微球蛋白［mg/L，*M*（*Q*_1_，*Q*_3_）］	3.27（1.94，5.23）	3.01（2.02，5.67）	3.65（2.43，6.60）	4.772	0.092
诱导后疗效评估［例（％）］				–	<0.001
sCR	37（41.1）	7（4.9）	2（1.1）		
CR	39（43.3）	76（53.5）	36（20.5）		
VGPR	12（13.3）	53（37.3）	88（50.0）		
PR	2（2.2）	4（2.8）	39（22.2）		
SD	0（0）	1（0.7）	3（1.7）		
PD	0（0）	1（0.7）	8（4.5）		

**注** Ig：免疫球蛋白；ISS：国际分期系统；R-ISS：修订的国际分期系统；HGB：血红蛋白；PLT：血小板计数；sCR：严格意义的完全缓解；CR：完全缓解；VGPR：非常好的部分缓解；PR：部分缓解；SD：疾病稳定；PD：疾病进展；–：部分分类变量采用Fisher精确检验，无*χ*^2^值

诱导治疗后ORR为96.8％（395/408），CR及以上疗效者197例（48.3％）。三组患者传统疗效的差异有统计学意义（*P*<0.001）：超深度阴性组、低负荷阳性组、高负荷残留组分别有76例（84.4％）、83例（58.5％）、38例（21.6％）疗效达CR及以上。197例疗效达CR及以上患者中，仍有61.4％（121/197）患者处于MRD阳性状态，其中38例CR患者的MRD负荷>10^−3^。而153例VGPR患者中12例（7.8％）MRD已达超深度阴性。CR及以上疗效患者中MRD达超深度阴性的比例显著高于VGPR患者（38.6％对7.8％，*P*<0.001）。

2. 生存分析：截至末次随访，中位随访49（14～74）个月，中位PFS期为54（3～76）个月。三组的PFS率及OS率均随着MRD负荷的降低而升高（*P*值均<0.001）（图1）。以高负荷残留组为参照组，低负荷阳性组及超深度阴性组疾病进展风险分别降低60％（*HR*＝0.40，95％*CI*：0.28～0.57）和86％（*HR*＝0.14，95％*CI*：0.08～0.26），超深度阴性组PFS率优于低负荷阳性组（*HR*＝0.36，95％*CI*：0.19～0.68，*P*＝0.005）。OS方面，低负荷阳性组（*P*＝0.027）及超深度阴性组（*P*＝0.001）死亡风险均较高负荷残留组显著下降，但两组间差异无统计学意义（*P*＝0.081）。MRD<10^−6^患者的疾病进展风险较MRD≥10^−6^且≤10^−5^患者降低（*HR*＝0.43，95％*CI*：0.20～0.92，*P*＝0.026），但OS的差异无统计学意义（*P*＝0.132）（图2）。

**图1:**
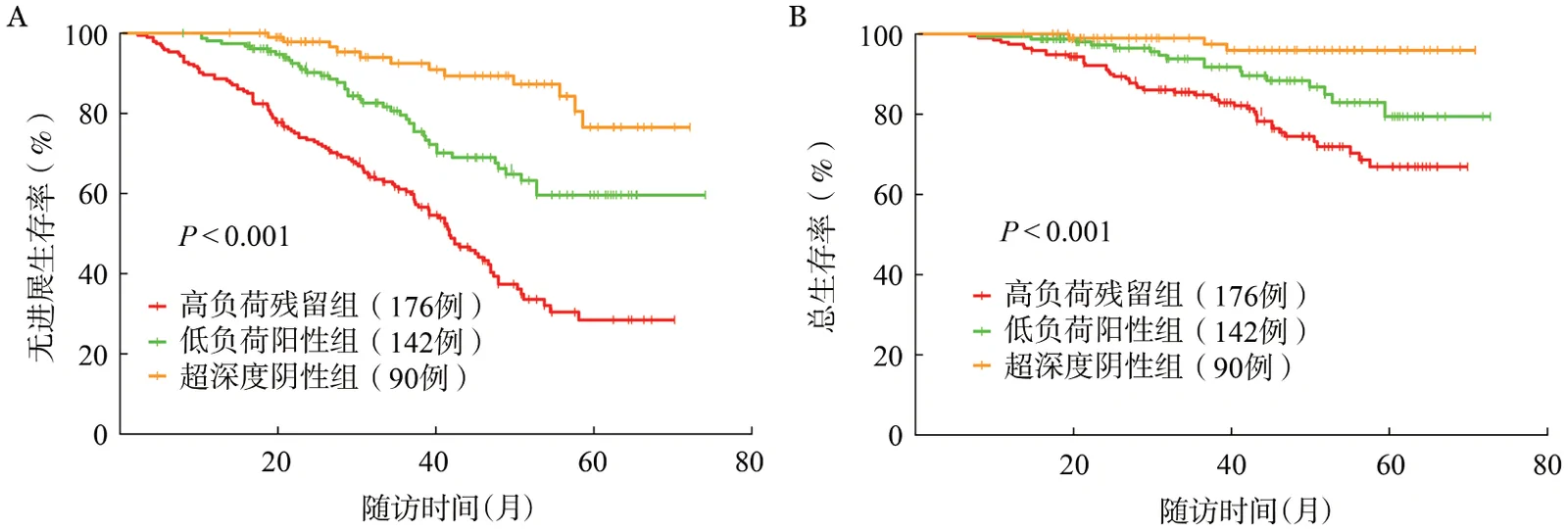
超深度阴性组、低负荷阳性组、高负荷残留组初治多发性骨髓瘤患者的无进展生存（A）和总生存（B）曲线

**图2:**
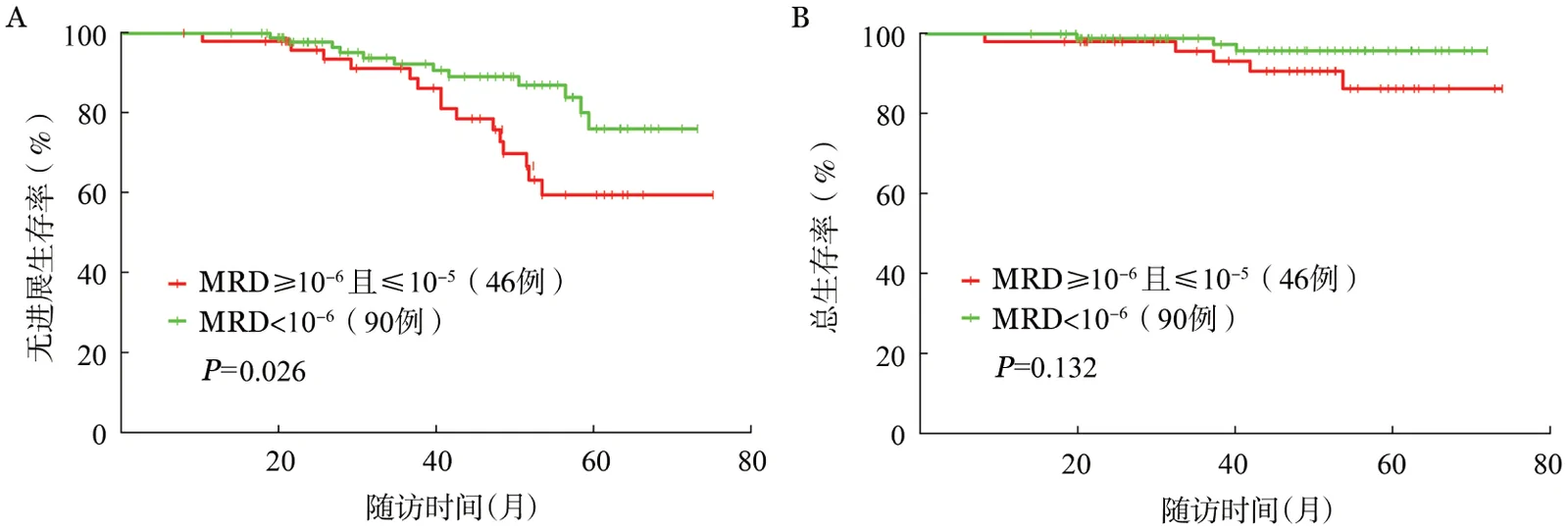
诱导治疗后微小残留病（MRD）≥10^−6^且≤10^−5^患者与MRD<10^−6^患者的无进展生存（A）和总生存（B）曲线

疗效达CR及以上的197例患者中，不同MRD负荷组PFS及OS的差异均有统计学意义（*P*值分别为<0.001和0.002）（**扫描本文首页二维码查阅附图1**）。以高负荷残留组为参照，超深度阴性组疾病进展风险降低87％（*HR*＝0.13，95％*CI*：0.06～0.28，*P*<0.001），与低负荷阳性组相比PFS获益亦显著（*HR*＝0.37，95％*CI*：0.18～0.78，*P*＝0.028）；OS分析显示，仅超深度阴性组患者死亡风险显著降低（*HR*＝0.13，*P*＝0.005）。在高危细胞遗传学及1q21阳性亚组中，不同MRD负荷组PFS和OS的差异均有统计学意义（*P*值均<0.05）（**描本文首页二维码查阅附图2、附图3**）。在t（11;14）亚组（45例）中，诱导治疗后超深度阴性组8例（17.8％），所占比例低于其他细胞遗传学异常亚组，且不同MRD负荷组间PFS（*P*＝0.058）及OS（*P*＝0.195）的差异均无统计学意义（**扫描本文首页二维码查阅附图4**）。

3. 基于竞争风险模型的极早期进展预测：Gray检验显示，三组CIF的差异有统计学意义（*χ*^2^＝72.5，*P*<0.001），高负荷残留组的CIF曲线始终高于其他两组（图3）。超深度阴性组、低负荷阳性组、高负荷残留组的24个月CIF分别为1.2％（95％*CI*：0.1％～5.9％）、8.3％（95％*CI*：4.4％～13.9％）、23.2％（95％*CI*：17.2％～29.7％），提示MRD负荷越高，早期进展风险越大（表2）。以高负荷残留组为参照，低负荷阳性组（*sHR*＝0.33，95％*CI*：0.22～0.50，*P*<0.001）及超深度阴性组（*sHR*＝0.15，95％*CI*：0.08～0.31，*P*<0.001）疾病进展风险均显著降低。超深度阴性组的疾病进展风险较低负荷阳性组降低（*sHR*＝0.48，95％*CI*：0.24～0.94，*P*＝0.032）。

**图3:**
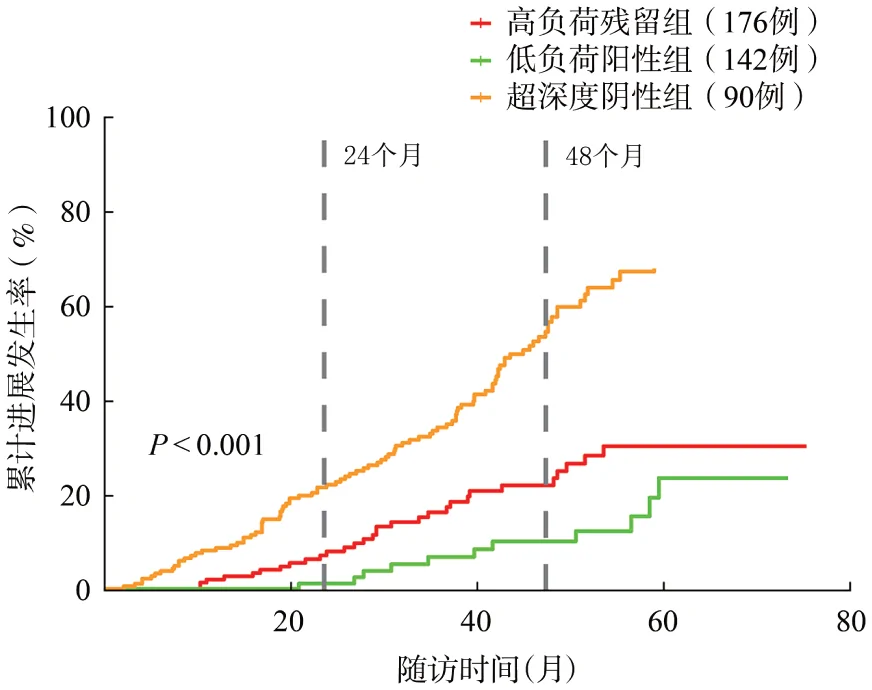
超深度阴性组、低负荷阳性组、高负荷残留组初治多发性骨髓瘤患者的累积进展发生率曲线

**表2:** 超深度阴性组、低负荷阳性组、高负荷残留组多发性骨髓瘤患者诱导治疗后累积进展发生率（CIF，％）

分组	24个月CIF（95％*CI*）	36个月CIF（95％*CI*）	48个月CIF（95％*CI*）
超深度阴性组（90例）	1.2（0.1～5.9）	7.1（2.6～14.8）	10.5（4.6～19.4）
低负荷阳性组（142例）	8.3（4.4～13.9）	17.0（10.8～24.5）	23.0（15.4～31.6）
高负荷残留组（176例）	23.2（17.2～29.7）	35.9（28.6～43.2）	59.3（50.2～67.2）

4. 预后因素分析：构建单因素及多因素Fine-Gray竞争风险回归模型评估MRD深度对疾病进展的独立预测作用，结果以森林图呈现（**扫描本文首页二维码查阅附图5**）。单因素分析显示，与超深度阴性组相比，高负荷残留组疾病进展风险显著升高（*sHR*＝6.37，95％*CI*：3.47～11.67，*P*<0.001）。高龄（*P*＝0.013）、R-ISS Ⅲ期（*P*＝0.046）、t（14;16）（*P*<0.001）及1q21获得/扩增（*P*＝0.002）亦为疾病进展的危险因素（表3）。将单因素分析中*P*<0.1的变量及重要协变量纳入多因素分析模型。MRD水平在校正年龄、分期及遗传学异常后仍为疾病进展的独立预测因素：与超深度阴性组相比，高负荷残留组疾病进展风险显著升高（*sHR*＝6.65，95％*CI*：3.58～12.33，*P*<0.001），低负荷阳性组风险亦显著升高（*sHR*＝2.18，95％*CI*：1.11～4.27，*P*＝0.024）。1q21获得/扩增（*sHR*＝1.98，95％*CI*：1.39～2.83，*P*<0.001）和t（14;16）（*sHR*＝1.78，95％*CI*：1.03～3.09，*P*＝0.038）亦为疾病进展的独立危险因素。

**表3:** 影响多发性骨髓瘤患者疾病进展的单因素和多因素分析

因素	单因素分析		多因素分析	
	*sHR*（95％*CI*）	*P*值	*sHR*（95％*CI*）	*P*值
年龄（≥65岁，<65岁）	1.65（1.11～2.44）	0.013	1.18（0.76～1.85）	0.460
R-ISS分期（Ⅲ期，Ⅰ/Ⅱ期）	1.53（1.01～2.32）	0.046	1.12（0.65～1.93）	0.690
del（17p）（阳性，阴性）	1.50（0.92～2.44）	0.110	1.37（0.78～2.42）	0.270
t（4;14）（阳性，阴性）	1.32（0.88～1.96）	0.180	1.40（0.88～2.23）	0.160
t（11;14）（阳性，阴性）	1.03（0.59～1.81）	0.920	1.02（0.58～1.80）	0.942
t（14;16）（阳性，阴性）	3.20（2.07～4.94）	<0.001	1.78（1.03～3.09）	0.038
1q21获得/扩增（阳性，阴性）	1.72（1.22～2.43）	0.002	1.98（1.39～2.83）	<0.001
巩固治疗（有，无）	1.15（0.82～1.62）	0.410	1.04（0.72～1.50）	0.850
维持治疗（双药，单药）	1.40（0.99～1.98）	0.057	1.39（0.98～1.98）	0.064
MRD水平（低负荷阳性组，超深度阴性组）	2.10（1.06～4.13）	0.032	2.18（1.11～4.27）	0.024
MRD水平（高负荷残留组，超深度阴性组）	6.37（3.47～11.67）	<0.001	6.65（3.58～12.33）	<0.001

**注** R-ISS：修订的国际分期系统；MRD：微小残留病

5. MRD深度与疾病进展的相关性：对138例疾病进展患者进行分析，三组患者比例的差异有统计学意义（*P*＝0.002）。高负荷残留组68.0％（66/97）表现为临床进展，其中12例伴髓外病变或浆细胞白血病样转化；低负荷阳性组临床进展比例为46.6％（14/30），16例表现为单纯生化进展；超深度阴性组中72.7％（8/11）为单纯生化进展，临床进展仅占27.3％（3/11）。进一步分析显示，高负荷残留组自诱导治疗结束至出现MM常见症状的中位时间为52（11～68）个月，较另外两组（均未达到）显著缩短（*P*<0.001）。

## 讨论

本研究基于NGS的三级MRD定量分层策略，突破了传统MRD阴性/阳性二分类评估的局限，在诱导治疗后这一更早时间节点实现了对早期进展风险的精细量化，为MRD由预后标志物向治疗决策工具转化提供了真实世界证据。现有研究多以<10^−5^作为MRD阴性判断标准，且多集中于auto-HSCT后单时间点二分类评估，基于竞争风险模型的早期定量进展风险分析尚匮乏。国内基于NGS（灵敏度10^−6^）的大样本真实世界研究证据亦十分有限，本研究可为该领域提供重要的循证依据。

早期MRD阴性状态的预后意义仍存在争议，多项临床试验将MRD阴性与预后良好联系起来[23]–[25]，但亦有研究对诱导治疗后早期MRD评估的预后价值提出质疑。Mohan等[23]的研究报道，早期获得MRD阴性反而与更高的复发率（50％对29％，*P*＝0.009）及MRD转为阳性风险（57％对38％，*P*＝0.020）有关。Tandon等[24]的研究表明，诱导治疗4个周期后疗效达VGPR与未达VGPR患者中位PFS期和OS期的差异均无统计学意义（PFS：31个月对29个月，*P*＝0.1；OS：89个月对91个月，*P*＝0.9）。上述研究提示，基于传统疗效标准或较低灵敏度MRD检测的早期评估可能不足以准确判断患者的长期预后风险。本研究采用高灵敏度NGS对诱导后MRD负荷进行三级定量分层，结果显示，超深度阴性患者在PFS、OS及疾病进展模式上均呈现显著优势，三组的差异有统计学意义，超深度阴性组疾病进展风险较低负荷阳性组降低64％。这一预后分层效能在传统CR患者、高危细胞遗传学及1q21阳性等亚组中也得到验证。Perrot等[26]基于IFM 2009试验证实，维持治疗阶段MRD阴性（<10^−6^）患者疾病进展风险降低78％。本研究在诱导治疗结束这一更早时间节点同样观察到超深度阴性的显著预后优势。另外，t（11;14）阳性患者诱导治疗后超深度MRD阴性率仅为17.8％，且不同MRD负荷水平患者PFS及OS的差异均无统计学意义。Liu等[27]的研究证实，t（11;14）单独存在组的MRD阴性率低于标准危险组，达到MRD阴性的时间亦显著延长，但MRD状态对该亚组的PFS及OS均无显著影响，呈现相对惰性的临床病程。因此，对t（11;14）亚型患者诱导治疗后的MRD评估结果应结合其独特遗传学背景解读。Paiva等[25]的前瞻性研究表明，采用高灵敏度检测方法时，诱导治疗后与移植后获得MRD阴性患者的生存结局几乎相同，进一步支持诱导治疗后MRD评估具有重要的临床价值。

传统疗效评估的灵敏度约为10^−2^，难以准确反映残留肿瘤负荷[12],[28]。国内研究报道，研究队列仅29.5％的患者达到严格意义的CR，而同期MRD阴性率却高达72.4％，提示传统疗效标准低估了真实缓解深度[29]。Munshi等[9]的研究显示，在疗效达到CR及以上的患者中，MRD阴性状态仍能显著改善PFS（*HR*＝0.38，95％*CI*：0.29～0.50，*P*<0.001）。本研究同样观察到这一现象：61.4％的CR患者仍处于MRD阳性状态，而7.8％的VGPR患者已实现超深度MRD阴性。CR亚组分析显示，超深度阴性组的疾病进展风险较高负荷残留组降低87％，证实了MRD检测的独立预后价值。

竞争风险模型分析显示，MRD负荷是影响早期疾病进展的重要因素。24个月时高负荷残留组的CIF（23.2％）约为超深度阴性组（1.2％）的19倍，多因素Fine-Gray模型进一步确认MRD深度是校正混杂因素后最强的独立预后因素（*sHR*＝6.65，*P*<0.001）。尽管接受了序贯auto-HSCT，高负荷残留组患者仍面临较高的早期进展风险，提示现有标准三联诱导治疗方案可能不足以完全克服其高危生物学特性。从MRD引导治疗的视角而言，诱导后MRD定量分层可为治疗强度的个体化调整提供客观依据，超深度阴性患者或可降低维持治疗强度以减少不良反应，而高负荷残留患者则可能从早期治疗升阶（如加入抗CD38单抗或嵌合抗原受体T细胞治疗）中获益，但上述策略均有待前瞻性随机对照研究加以验证。

本研究队列1q21获得/扩增发生率（53.7％）明显高于西方队列报道的34％～37％，与中国NDMM患者的流行病学特征相符[30]–[31]。Pasvolsky等[32]报道，移植后MRD阴性患者的PFS显著优于MRD阳性者，但1q21扩增（≥4个拷贝）仍是PFS的独立不良预后因素（*HR*＝2.03，*P*＝0.003）。与本研究多因素分析结果相似，1q21异常在MRD分层基础上仍具有独立的预后价值。

MRD负荷亦与复发时的疾病侵袭性密切相关。研究发现，高负荷残留组68.0％患者发生临床进展，超深度阴性组72.7％的进展仅表现为单纯生化进展。Goldman-Mazur等[33]报道，生化进展组的二线治疗中位持续时间（17.0个月对9.6个月）及复发后中位OS期（59.4个月对26.2个月）均优于临床进展组（*P*值均<0.001）。此外，Mohan等[23]的研究也发现，MRD转为阳性患者的临床复发率（53％对4％，*P*<0.001）和生化复发率（20％对0％，*P*<0.001）均显著高于持续阴性者，从动态监测角度进一步支持了MRD阴性的预后价值。

本研究存在以下局限性：单中心回顾性设计存在选择偏倚；MRD仅为诱导治疗后单一时间点评估，MRD动态转变对长期预后的影响有待后续研究进一步阐明。髓外病变缓解状态主要依据低剂量CT联合全身骨显像结果判定，PET-CT的缺失可能低估部分患者髓外疾病负荷。本研究中超深度阴性组与低负荷阳性组OS的差异无统计学意义，可能与随访时间不足有关，有待更长随访时间的前瞻性研究验证。

综上，基于NGS（灵敏度10^−6^）的诱导治疗后MRD定量分层是NDMM早期进展的独立预测因素，其预后分层效能显著优于传统疗效指标，可为临床个体化风险分层与早期治疗决策优化提供重要循证依据。高负荷残留患者的强化治疗策略有待前瞻性研究进一步探索。

## Supplementary Material


